# Supplementary figures and images for: Single-cell transcriptomics reveals expression profiles of Trypanosoma brucei sexual stages
Source: PLoS Pathog. 2022 Mar 7;18(3):e1010346. doi: 10.1371/journal.ppat.1010346 (PMC8939820; doi:10.1371/journal.ppat.1010346)

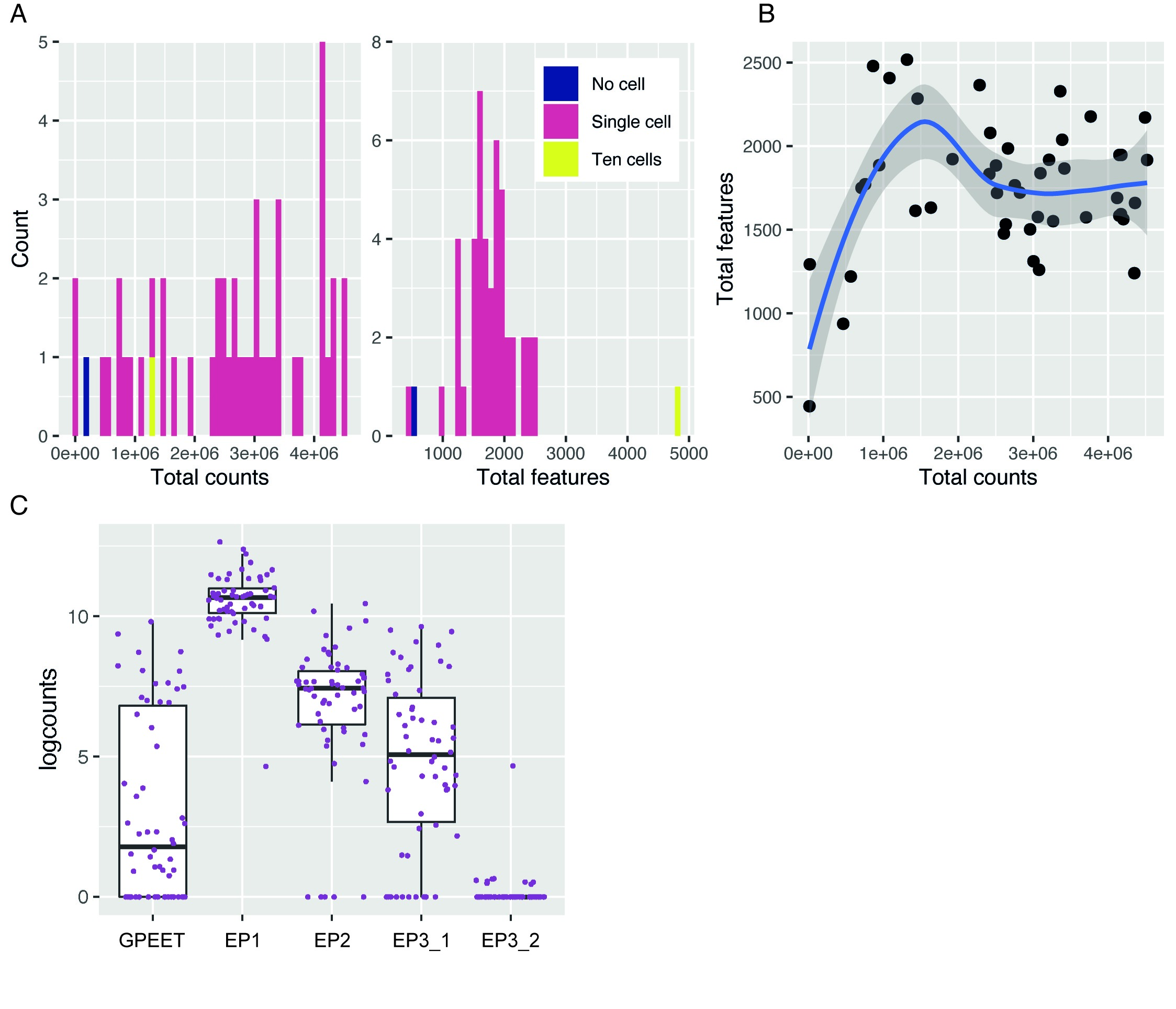

Supplement: S1 Fig — Forty-eight transcriptomes were generated using Smart-seq2 from parasites in a procyclic culture including a no cell and ten cell control. (A) The distribution of the total counts and total features (genes) detected in these 48 transcriptomes. (B) The total features plotted against total counts for the 46 single-cell transcriptomes shows a plateau as features and counts increase, suggesting that sequencing was saturated for these cells. We detected a mean of 2.6x10^6^ reads and 1756 features per single-cell transcriptome. (C) Expression of procyclic surface antigen genes GPEET (Tb927.6.510), EP1 (Tb927.10.10260), EP2 (Tb927.10.10250), EP3_1 (Tb927.6.520), EP3_2 (Tb927.6.480). (TIF) [file ppat.1010346.s001.tif]

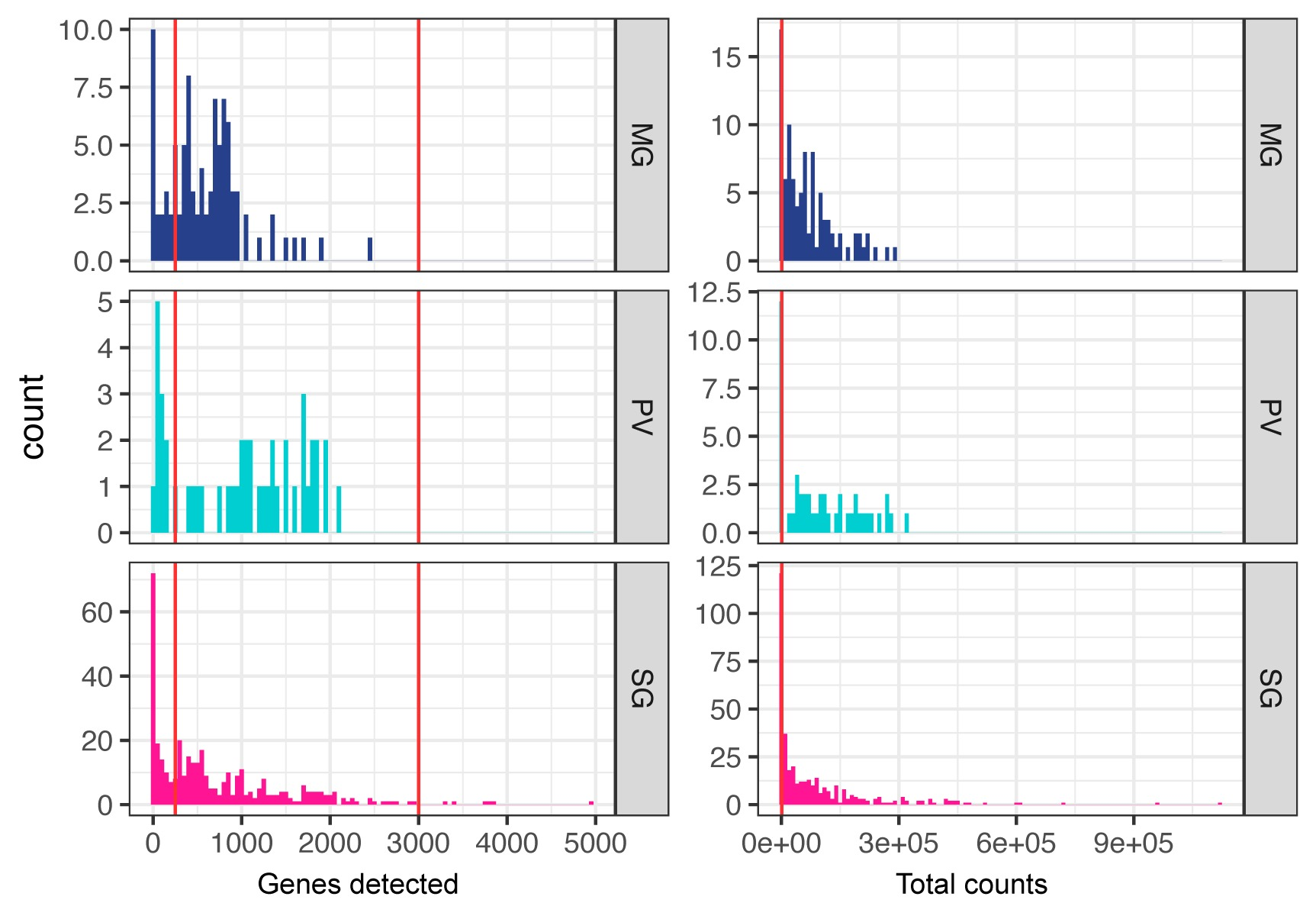

Supplement: S2 Fig — The distribution of genes detected (left) and counts (right) in each cell across the three insect tissues: midgut (MG), proventriculus (PV), and salivary glands (SG). Cells with fewer than 40 or more than 3000 genes per cell were removed. Additionally, cells with fewer than 1000 reads were removed. Cut-offs are represented by the red vertical lines in each histogram. After QC we detected a mean of 889 genes per cell and 1.1x10^5^ counts per cell. (TIF) [file ppat.1010346.s002.tif]

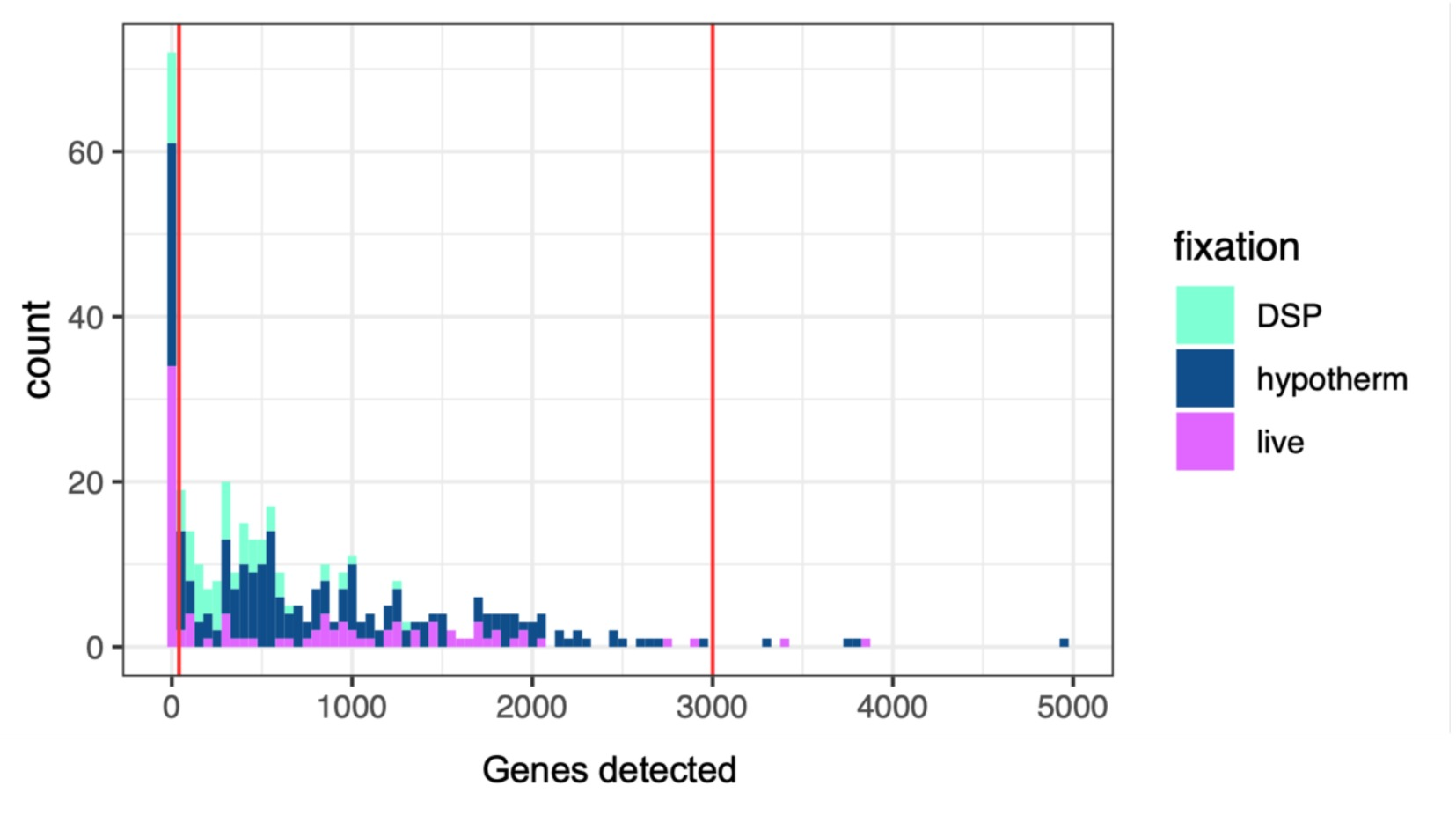

Supplement: S3 Fig — The distribution of features detected in salivary gland cells across the two preservation treatments (DSP and hypothermosol) compared to live parasites. Although there were slight differences in detection between the different treatments, caution must be taken in interpreting these differences as the fixation methods are confounded with the different timepoints collected (DSP: day 40; hypothermosol: day 24; live: day 21). (TIF) [file ppat.1010346.s003.tif]

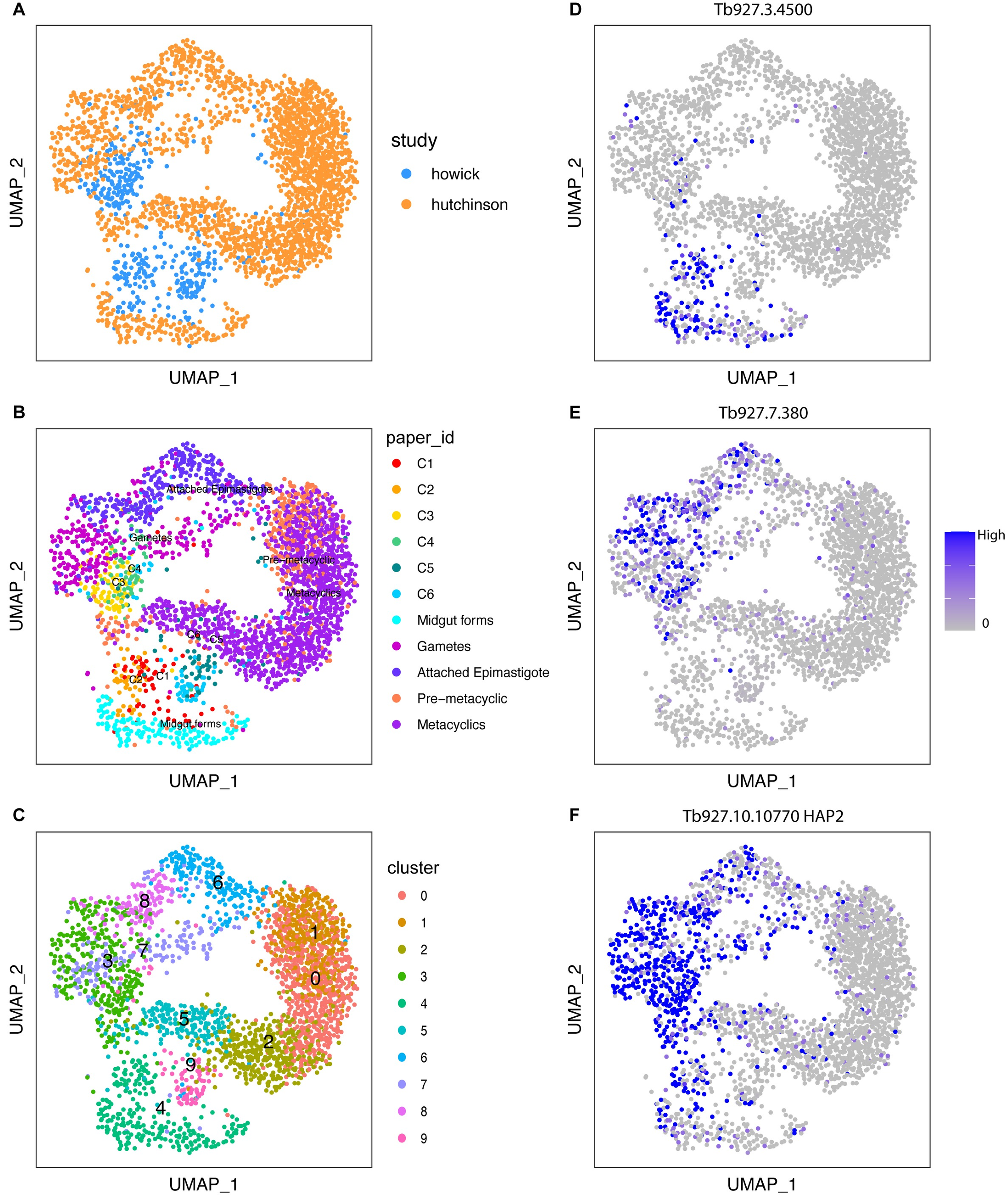

Supplement: S4 Fig — All 388 tsetse transcriptomes were integrated with the Hutchinson dataset collected from salivary glands [20] using Seurat’s data integration function. Plots show the UMAP of integrated data coloured by study (A), cluster identity from the different studies (paper_id) (B), integrated cluster assignment (C), or gene of interest (D-F). FHc (Tb927.3.4500) was the top marker gene (based on adjusted p-value) for the midgut and proventricular form cluster 4 (D). Tb927.7.380 (hypothetical protein, conserved) was the top marker gene for cluster 3 which contained gamete and epimastigote forms. HAP2 (Tb927.10.10770) (F) was not a marker gene for the gamete cluster likely because of its ubiquitous expression across non-metacyclic forms. Although we were able to identify conserved marker genes across the two studies, separation remained in the UMAP for all cell-types (A-B) and only the non-metacyclic forms co-clustered across the two studies and only at a granular level. The metacylic forms likely did not cluster together because of different VSG repertoires, and the separation across other cell-types may be due to time point, strain-specific expression patterns, or collection methods. Conserved marker genes for clusters 3 and 4 can be found in S3 Table. (TIF) [file ppat.1010346.s004.tif]

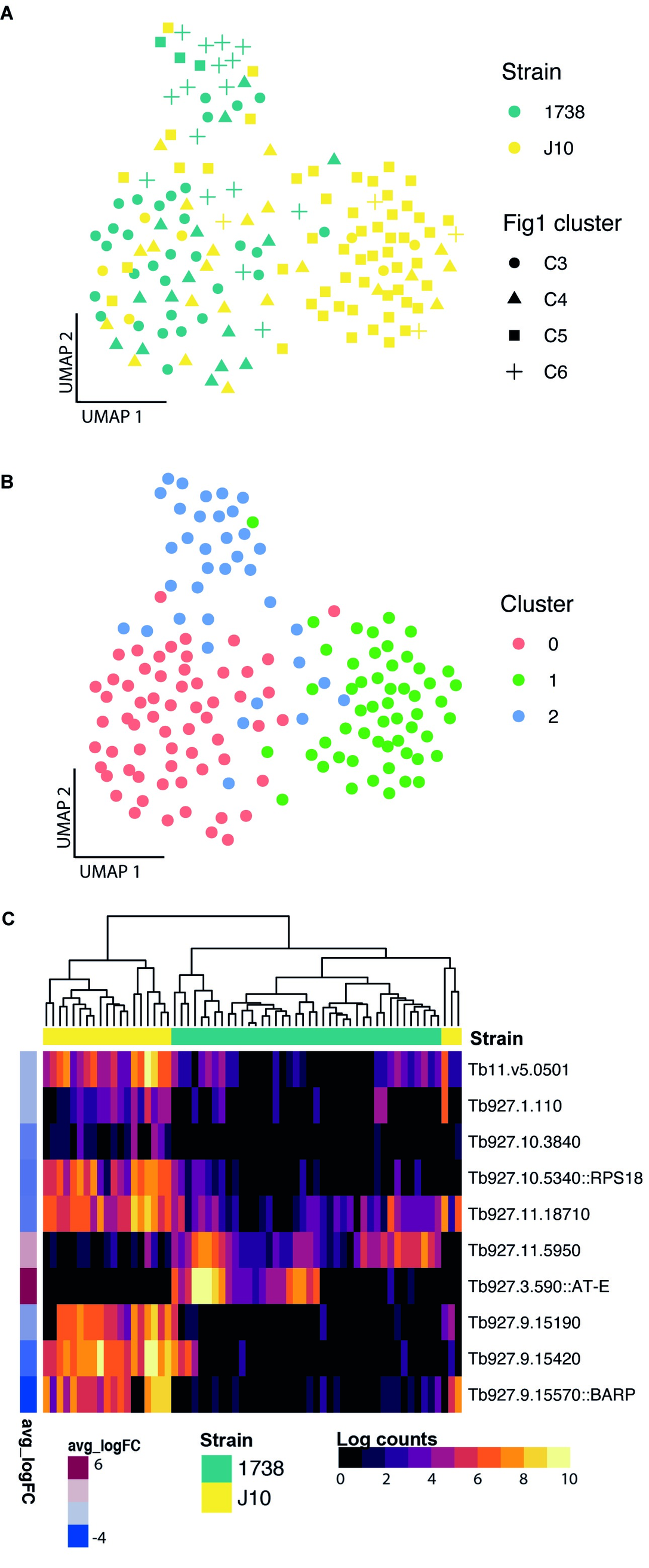

Supplement: S5 Fig — Strain-specific gene expression (A). A UMAP of the day 24 SG parasites integrated by strain (1738 and J10). Points are coloured by strain and shaped by Fig 1 cluster. (B). The integrated UMAP coloured by new cluster from the integration analysis. Cluster 0 has a representation of both strains, whereas cluster 1 and 2 are composed primarily of strain 1738 or J10, respectively. (C) Differential expression was performed between strains within cluster 0. The ten genes differentially expressed between the two strains are displayed on a heatmap. (TIF) [file ppat.1010346.s005.tif]

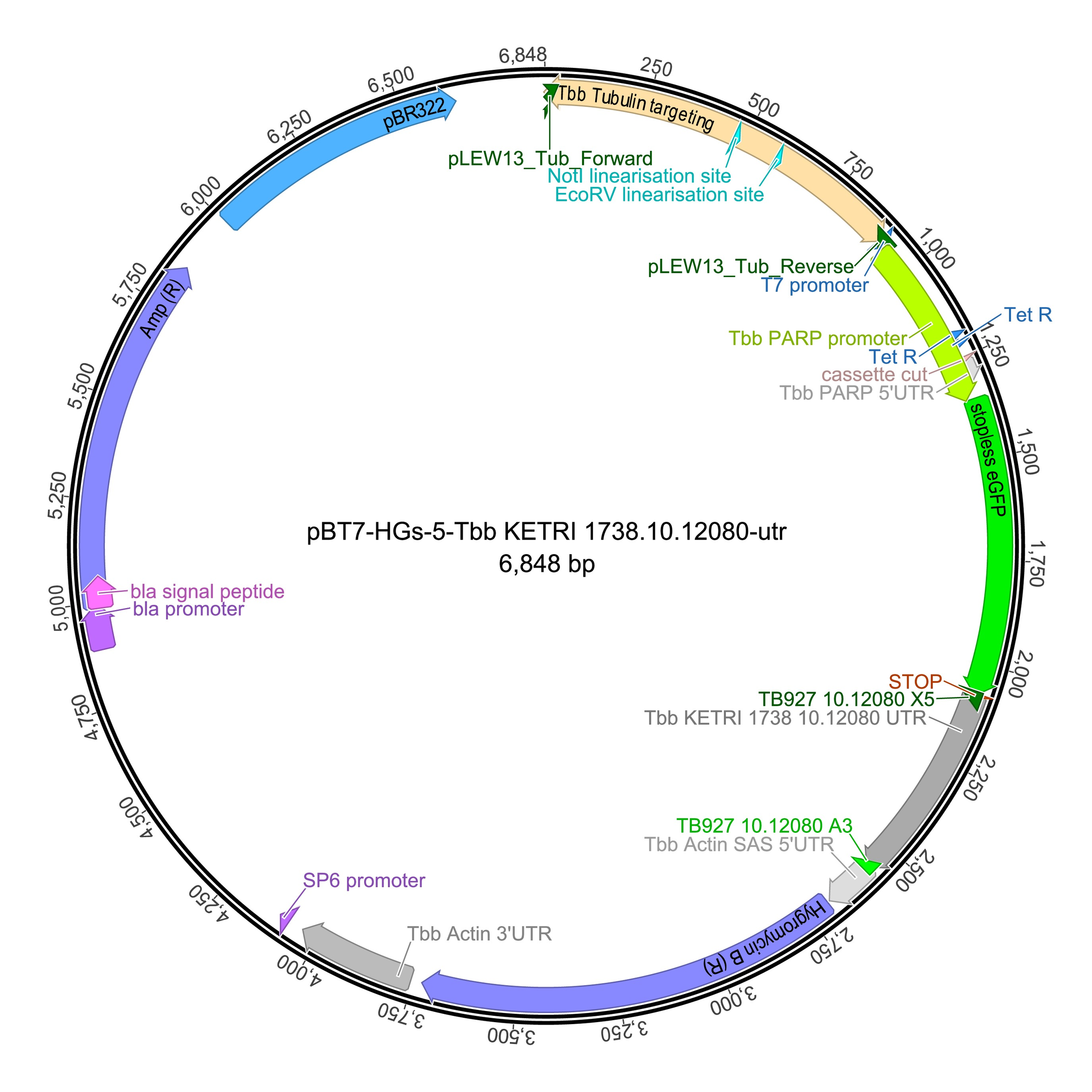

Supplement: S6 Fig — Life cycle selective expression of Tb927.10.12080 was investigated through a reporter construct where the expression of GFP was controlled by ~500 bp of UTR downstream of the gene. For this study a stable transformant line was generated in strain 1738 using the 3’ UTR from its endogenous gene and integrated into the tubulin locus. (TIF) [file ppat.1010346.s006.tif]
