# Supplementary material for: Single-cell transcriptomics reveals expression profiles of Trypanosoma brucei sexual stages
Source: PLoS Pathog. 2022 Mar 7;18(3):e1010346. doi: 10.1371/journal.ppat.1010346 (PMC8939820; doi:10.1371/journal.ppat.1010346)
Supplement: S7 Table — (DOCX) [file ppat.1010346.s013.docx]

| **Cell Type** | **Fluorescent** | **Non-Fluorescent** | **Total Cells** |
| --- | --- | --- | --- |
| Epimastigote/Dividing epimastigote | 0 (0%) | 86 (100%) | 86 |
| Trypomastigote/Dividing trypomastigote | 0 (0%) | 24 (100%) | 24 |
| Asymmetric Divider | 0 (0%) | 19 (100%) | 19 |
| Pre-metacyclic/Dividing pre-metacyclic | 0 (0%) | 29 (100%) | 29 |
| Metacyclic | 0 (0%) | 3 (100%) | 3 |
| Trypomastigote-Epimastigote divider | 0 (0%) | 4 (100%) | 4 |
| Meiotic Divider | 3 (10%) | 26 (90%) | 29 |
| Meiotic intermediate 3N | 5 (50%) | 5 (50%) | 10 |
| Meiotic intermediate 2N | 28 (80%) | 7 (20%) | 35 |
| Meiotic intermediate 3K1N or 4K1N | 38 (83%) | 8 (17%) | 46 |
| Meiotic intermediate - final division to two gametes | 34 (79%) | 9 (21%) | 43 |
| 1K1N Gamete | 11 (65%) | 6 (35%) | 17 |
| 2K1N Gamete | 19 (86%) | 3 (14%) | 22 |
| Unidentifiable | 44 (45%) | 53 (55%) | 97 |
| **TOTAL** | **182 (39%)** | **282 (61%)** | **464** |
