# Supplementary material for: Single-cell transcriptomics reveals expression profiles of Trypanosoma brucei sexual stages
Source: PLoS Pathog. 2022 Mar 7;18(3):e1010346. doi: 10.1371/journal.ppat.1010346 (PMC8939820; doi:10.1371/journal.ppat.1010346)
Supplement: Supplementary file 8 [file ppat.1010346.s012.docx]

Cell type	GPEET- GFP				EP-RFP			Total	Notes
	No	V.faint	Faint	Yes	No	Faint	Yes	cells	
1K1N gamete		2	5	8	0	0	15	15	all GPEET&EP **Total gametes:**
2K1N gamete	2	3	3	11			19	19	82% GPEET&EP **90% GPEET&EP, 10% only EP**
2K1N meiotic		1		1			2	2	all GPEET&EP
3K1N				3			3	3	
4K1N				1			1	1	**Total intermediates:**
1K2N		2		2			4	4	**1/22 (5%) only EP;**
2K2N		1		3			4	4	**21/22 (95%) GPEET&EP**
3K2N	1	1		5			7	7	
4K2N				1			1	1	
1K3N				1			1	1	
3K3N				1			1	1	
1K1N epi	2		2	2	1		5	6	1/6(17%)none;1/6(17%)EP;4/6(66%)GPEET&EP
1K1N short epi			1			1	0	1	GPEET&EP
1K1N trypo	3	1	1	1			6	6	3/6 (50%) only EP; 3/6 (50%) GPEET&EP
2K2N div trypo	1				1		0	1	no GPEET or EP
1K1N meta	6				6		0	6	no GPEET or EP
Total	15	11	12	40	8	1	69	78	

Cell type	BARP- GFP				EP-RFP			Total	Notes
	No	V.faint	Faint	Yes	No	V.faint	Yes	cells	
1K1N gamete	4		1	10			15	15	73% BARP&EP **Total gametes:**
2K1N gamete	4	3	2	9			18	18	78% BARP&EP. **76% BARP&EP, 24% only EP**
2K1N meiotic	1		2	2			5	5	80% BARP&EP
3K1N	1	2					3	3	
1K2N	2		1	3			6	6	**Total intermediates:**
2K2N	1	2	4	5			12	12	**7/36 (19%) only EP;**
3K2N	2			1			3	3	**29/36 (81%) BARP&EP**
4K2N		1					1	1	
5K2N			1	1			2	2	
2K3N				2			2	2	
3K3N		1	2	2			5	5	
4K3N	1			1			2	2	
1K1N epi		1	2	6			9	9	BARP&EP
2K1N div epi				3			3	3	BARP&EP
2K2N div epi	2	1		3			6	6	2/6 (33%) only EP; 4/6 (67%) BARP&EP
1K1N trypo	2	2	1	2			7	7	2/7 (29%) only EP; 5/7 (71%) BARP&EP
1K1N pre-meta			1	1	1	1	0	2	1/2 (50%) only BARP; 1/2 (50%) BARP&EP
1K1N meta	8				8		0	8	no BARP or EP
Total	28	13	17	51	9	1	99	109	
