# Supplementary material for: Single-cell transcriptomics reveals expression profiles of Trypanosoma brucei sexual stages
Source: PLoS Pathog. 2022 Mar 7;18(3):e1010346. doi: 10.1371/journal.ppat.1010346 (PMC8939820; doi:10.1371/journal.ppat.1010346)
Supplement: Attachment — Submitted filename: ResponseToReviews.docx [file ppat.1010346.s015.docx]

Dear Editors,

Please find our response to editorial and reviewer comments below in blue. Thank you for taking time to improve our manuscript.

Sincerely,

Virginia Howick

**Response to reviews of “Single-cell transcriptomics reveals expression profiles of *Trypanosoma brucei* sexual stages”**

**Editor**

I am returning your manuscript with three reviews. After reading the manuscript and the reviews, all the comments can be addressed **without further experimentation**. However, several points highlighted by the reviewers must be addressed.

Please pay particular attention to the language used to describe the role of Tb927.10.12080.
Please consider the addition of data mentioned in the materials and methods to Figure 6 to further strengthen the interpretation of the role of Tb927.10.12080.
All three reviewers have highlighted sections in the text that need to be expanded for clarity or typos addressed

These revisions must be addressed in order to prepare this manuscript for publication.

**We thank the editor for taking the time to handle our manuscript. We have modified the language used regarding the potential role of Tb927.10.12080 and have also added data to Figure 6. We have edited the other sections of the manuscript as the reviewers have suggested and describe the details of these modifications below.**

**Reviewer's Responses to Questions**

**Part I - Summary**
Please use this section to discuss strengths/weaknesses of study, novelty/significance, general execution and scholarship.

Reviewer #1: The manuscript by Howick et al., “Single-cell transcriptomics reveals expression profiles of Trypanosoma brucei sexual stages”, is an important contribution to the field of trypanosome development in the tsetse vector. The authors define and characterize different life-cycle stages transcriptomes, and, combined with previous studies using scRNAseq improve our understanding of the intricate development of T. brucei. The available repertoire of metacyclic VSGs (mVSGs) is expanded by 11 new genes which the authors identified in the cell lines used. The focus of the study is the sexual development of T. brucei, a process which is still not well understood mechanistically. A new marker for meiotic stages was identified and verified using a fluorescent reporter. The manuscript is well-written and is an important step to understanding meiosis and sexual reproduction in T. brucei, as well as its evolution in eukaryotes.

Reviewer #2: In this manuscript, Howick et al use single cell transcriptomics to study sexual recombination in African trypanosomes. The authors obtained single cell transcriptomes for trypanosomes from the midgut (MG), proventriculus (PV) and salivary gland (SG) of infected tsetse flies, covering the major stages of development in the insect host. The range of data, covering three organs, attached and free SG parasites, multiple strains, experimental crosses, assembly of metacyclic VSG expression sites, and the first description of the surface coat of meiotic and gamete cells is impressive.
These data therefore go further than the recent salivary gland single cell transcriptomes from Vigneron et al (2020) and Hutchinson et al (2021), which focused on the salivary glands only. The focus on sexual stages in this manuscript gives important novelty over these previously published transcriptomes.
This manuscript should be published without further experimentation. It will catch the attention not only of parasitologists, but also of people interested in the history of sexual recombination. We believe it could benefit from the addition of some data (from existing experiments but not shown), and of a more elaborate discussion. This one is a bit short but this is probably a consequence of the results and discussion being combined in a single section.

Reviewer #3: The manuscript describes single cell transcriptomes of trypanosomes from the salivary glands of tsetse flies. The findings are interesting and are confirmed by both the know biology in the salivary gland and by experimentation. A good read.

**We sincerely thank all the reviewers for their encouraging words and overall positive views of the manuscript.**

**Part II – Major Issues: Key Experiments Required for Acceptance**
Please use this section to detail the key new experiments or modifications of existing experiments that should be absolutely required to validate study conclusions.

Generally, there should be no more than 3 such required experiments or major modifications for a "Major Revision" recommendation. If more than 3 experiments are necessary to validate the study conclusions, then you are encouraged to recommend "Reject".

Reviewer #1: Major points:

My main concern stems from the use of the following phrases to describe Tb927.10.12080:
p. 35 “We speculate that this protein… plays a role in gamete formation and/or fusion.”
p. 369 “…we hypothesized that this gene may play a role in sexual development.”
p. 394 “…it could play a vital role in processes that allow for genetic exchange in T. brucei…”
p. 421 “We speculate that this protein… plays a role in gamete formation and/or fusion.”
The results presented clearly demonstrate that the protein is expressed and likely functions during these developmental stages. However, speculating that this gene plays a direct role in the process is not justified. To demonstrate a functional role for Tb927.10.12080 in the sexual development of T. brucei the authors can use the same plasmid vector they used for the fluorescent reporter, but instead of the reporter, have a long hairpin construct for RNAi targeting the gene transcript. Constitutive expression should not be a problem because the protein does not seem to be expressed in other life-cycle stages in the fly. If RNAi against Tb927.10.12080 interferes with meiosis, scored by cell morphology/KN counts, the authors will have a very strong case for a direct role of this gene in T. brucei sexual development.
Alternatively, the language can be changed to indicate that there is a distinction between “functioning during” and “playing a role in”.

**We have updated the text to clarify (line 35 and 394) that further experiments are required to establish whether the protein plays a role in gamete formation or fusion.**

With the identification of additional 11 mVSG sequences to complement the set of already available metacyclic VSGs from different T. brucei cell lines, have the authors identified any features that distinguish these VSGs from the ones found in bloodstream expression sites? It is an important general question that still awaits clarification. The predominant current view is that they are indistinguishable from each other, however, it is important to comment on this point.

**The mVSG sequences were indistinguishable from the BSF VSGs identified in Kay et al 2021. As with previously identified mVSG, the expression sites (and pattern of expression observed) were the genomic feature that distinguished these 11 mVSG from BSF VSGs.** **We have uploaded an additional review file containing a (large) tree of all VSG from these strains identified in Kay et al 2021 (in review PloS path) and highlighted the location of the mVSG.**

There should be a slightly expanded discussion/interpretation of the results indicating that GPEET procyclin is highly expressed in meiotic intermediates and gametes. GPEET is a presumed marker for early procyclics, as the authors point out. Or is this notion incorrect, according to the results presented here?

**Our data support an additional role of GPEET outside of the early procyclic forms where expression is coming back on after a decrease in late procyclic and proventricular parasites. We have added the following paragraph to the results/discussion section (line 365).**

**“Although previous work has shown that *GPEET* and *EP* transcripts are present in salivary gland parasites, expression of the corresponding proteins have not been demonstrated [44]. Instead, BARPs have been considered the major surface proteins in non-metacyclic, salivary gland forms [7]. Our data support a model where epimastigotes and sexual forms are expressing a diversity of surface proteins at high levels including BARPs, GPEET and EP. Additionally, the expression of these procyclins outside the midgut indicates they might play a functional role in these stages as well, and further experiments are needed to understand if they are required for development or interactions of meiotic dividers and gametes.”**

Reviewer #2: No new experiments required

Reviewer #3: In places the manuscript will be difficult to follow without knowledge of the software, there are a couple of instances of software being used without providing a few words on what type of analysis it does. It could be made more accessible for a wider audience.

**We have updated the methods section so that there is a short description of what each software does and have provided the corresponding references for readers that are interested in the details of how each software we used works.**

Could the authors provide an estimate of the time from removing the cells from their natural habitats and lysing / fixing them for RNA preparation? As the mean half life of mRNAs is ~20 minutes in trypanosomes, a consideration of / comment on any possible effect of the cell handling on transcriptome would be helpful.

**Live cells were processed in less than 1 hour and then stored on ice or fixed prior to sorting. Transport to the sorting facility and preparation of the sorter took up to five hours. Processing, storage, and fixation could all have an impact on the cell quality and transcript detection; however, we were unable to fully assess this in the paper because storage and handling varied by cell types (eg the attached epimastigotes required disassociation prior to fixation, whilst the other cells did not undergo this treatment). Based on the data we do have, we think that cell-type is a greater driver of transcript detection compared to handling (eg the metacyclics had the fewest genes per cell and are thought to be more transcriptionally quiescent as they wait for release into the next host). Further work is needed to optimise fixation for single-cell transcriptomics in parasites to allow for high quality data from field samples and greater flexibility in handling times.**

**Part III – Minor Issues: Editorial and Data Presentation Modifications**
Please use this section for editorial suggestions as well as relatively minor modifications of existing data that would enhance clarity.

Reviewer #1: Minor point:

1. In Fig. 2A, the color of the VSG box on the left and the colors of the VSG boxes in the contigs should be synchronized. They should either be all white (the color is perhaps not needed in this panel), or the one on the left should reflect the range of colors used.

**We have updated Figure 2A so that the outline of the box matches the legend (solid black outline) for the VSGs. We have left the fill with the various colours so that the reader can match each VSG genomic context, with expression levels across the single cells shown in Fig 2D. We have clarified this in the figure legend. We have also made the VSG rectangle thinner so that it is not confused with the ESAGs.**

2. “as LRRs are protein recognition motifs, this could be significant in gamete interactions.” This is highly unlikely because published data indicate that these specific proteins are highly enriched in the nucleus.

**We thank the reviewer for pointing this out. After further investigation of localisation data from Goos et al (PMID: 28727848) and Tryptag, we have decided to remove this sentence. Of the LRRs Tb927.9.14570 was not listed in Goos et al and there is no localisation data; Tb927.8.7180 shows ~25% cells with nuclear localisation and ~75% cytoplasmic on Tryptag and is listed in Goos et al 2017 as nuclear enriched expression in procyclics; Tb927.7.7110 shows weak cytoplasmic localisation in Tryptag and is not listed in Goos et al. Although there is some variation in localisation data, lack of surface expression of these proteins in other stages indicates that they likely don’t play a role in gamete interactions.**

Reviewer #2: Main comments
The discussion of the sexual recombination between strains 1738 and J10 is somewhat brief, with several unanswered questions:
1) at which point in the Pseudotime analysis are the hybrid transcriptomes found? Can we reliably obtain a temporal order of events from these data, i.e., does gametocytogenesis precede epimastigote attachment to the epithelium?

**As the pseudotime analysis shown in Figure 3 only includes the 1738 cells where we have a greater range of time points sampled, we do not have an exact pseudotime value for the hybrid cells identified in Figure 4. However, based on the UMAP and clustering analysis we can see that the majority of the hybrids are falling into C4 with one in C6 and two in C3, suggesting that gametocytogenesis precedes epimastigote attachment, which we think occurs at the branch between the two clouds of cells (fig 3). However, we are working with very small numbers of cells for both the hybrids and the attached cells that appear to be epimastigotes, so it is difficult to say with certainty exactly what the sequence of events is.**

2) Were the authors able to understand how meiotic recombination alters the distribution of SNPs throughout the chromosomes of the hybrids?

**This question is currently being addressed in Kay et al (2021, *in review*, PLoS Pathogens), which contains the full genome data of the hybrids and both parental strains. Given the sparse nature of single-cell data, it is difficult to assess patterns of recombination because of lack of coverage.**

The expression analysis of Tb927.10.12080 is interesting and seems solid. The experimental design could impact interpretation, since a procyclin promoter is used to drive expression of the GFP reporter. We believe that from the description of the experiment in the materials and methods, the authors have images of midgut parasites showing that GFP is not expressed. These are an important control to illustrate that the regulation observed is indeed controlled by the 3´ UTR rather than the procyclin promoter and could be added to strengthen Figure 6.

**We have added control images from the proventriculus to Figure 6B to illustrate that the regulation observed is not being driven by the procyclin promoter as the reviewers have suggested.**

Minor comments

Line 103 and legend figure S1. Is it 46 or 48 cells? The number differs at several places of the text. Please correct.

**We sequenced 48 wells from a 96-well plate. 46 contained single-cells, and the other two controls contained no cells and ten cells. We have checked the numbers mentioned throughout the manuscript.**

Line 117. “A further 62 1738 parasites” is a bit confusing. We would suggest using “strain 1738” or “strain J10” throughout the manuscript.

**We have added “strain” to J10 and 1738 to clarify throughout the manuscript.**

Line 130-140, the description of the number of transcriptomes was a little hard to follow. Could the authors inset a table into Fig 1A to help the reader?

**We have added the number of cells from each collection to the schematic in Fig 1A and rewritten the section to make it clearer.**

Figure 1. The clusters at Fig. 1D are difficult to follow, especially since they are not presented in the same order as at Fig. 1C, which is a bit confusing. We suggest adding extra labels or keeping the same order.

**We have reordered the clusters on the heatmap to match Fig 1C.**

Page 13: morphological features used for gamete identification were not very clear. Presumably, this is based on previous papers, but we could not find information in the manuscript.

**We have added specifics on how the morphological forms were identified to the section describing Figure 5 on page 13 (meiotic forms = 2K1N cell with large posterior nucleus and two flagella; gametes = 1K1N or 2K1N cells with small pear-shaped body and relatively long anterior flagellum) and have added the citation describing this work.**

Give a reference to the TrypTag paper for readers not specialised in trypanosomes (Halliday et al MBP2019).

**We have added the TrypTag references.**

Figure S3, should the x-axis label be “Genes detected”?

**We have changed the axis label as suggested.**

Line 239 “HMM” acronym not described

**We have changed HMM to hidden Markov model.**

Line 290, no citation for Slingshot (though it appears in M&M)

**We have added to citation for Slingshot to results section as well.**

The code used to analyze the data is not available at the stated URL.

**We have updated the Github with code and expression matrices.**

Sebastian Hutchinson and Philippe Bastin, ESPCI & Institut Pasteur, Paris

Reviewer #3: There are a small number of typos, eg line 100 should be RBP6 not RBP 6

**We have carefully read over the MS for typos**
